# A combined human case of *Dirofilaria ursi* infection in dorsal subcutaneous tissue and *Anisakis simplex* sensu stricto (s.s.) infection in ventral subcutaneous tissue

**DOI:** 10.1186/s41182-017-0067-4

**Published:** 2017-11-01

**Authors:** Minoru Yamada, Namiko Shishito, Yoshihiro Nozawa, Shigehiko Uni, Keisuke Nishioka, Takaaki Nakaya

**Affiliations:** 1 Department of Infectious Diseases, Kyoto Prefectural University of Medicine, Kyoto, Japan; 2 Department of Pathology, Shirakawa Kosei General Hospital, Fukushima, Japan; 3 Institute of Biological Sciences, Faculty of Science, University of Malaya, Kuala Lumpur, Malaysia

**Keywords:** Helminthic subcutaneous infections, *Dirofilaria ursi*, *Anisakis simplex* sensu stricto (s.s.), A combined human case

## Abstract

**Background:**

*Dirofilaria ursi* is a filarial nematode that parasitizes the subcutaneous tissues of the American black bear (*Ursus americanus*) and Japanese black bear (*Ursus thiabetanus japonicus*). *D*. *ursi* that has parasitized black bears has the potential to subsequently infect humans. In addition, extra-gastrointestinal anisakiasis is less common in Japan.

**Case presentation:**

We report a case of ventral subcutaneous anisakiasis and dorsal subcutaneous dirofilariasis that was acquired in Fukushima, in the northern part of Japan. The patient was an 83-year-old Japanese female, and subcutaneous parasitic granulomas were present on her left abdomen (near the navel) and left scapula. A pathological examination of the surgically dissected tissue sections from each region demonstrated eosinophilic granulomas containing different species of parasites. To enable the morphological and molecular identification of these parasites, DNA was extracted from paraffin-embedded sections using DEXPAT reagent, and the cytochrome oxidase 2 (COX2), internal transcribed spacer 1 (ITS1), 5.8S and ITS2 regions of the *Anisakis* larvae, and the 5S rRNA region of the male *Dirofilaria* were sequenced. The PCR products were examined and compared with DNA databases. Molecular analysis of the COX2 and 5S rRNA sequences of each worm revealed that the nematode found in the ventral region belonged to *Anisakis simplex* sensu stricto (s.s.) and the male *Dirofilaria* found in the dorsal region was classified as *D. ursi*.

**Conclusion:**

The present case showed a combined human case of *D. ursi* and *A. simplex* s.s. infections in subcutaneous tissues. The results of this study will contribute to the identification of unknown parasites in histological sections.

## Background

*Dirofilaria ursi* is a filarial nematode that parasitizes the subcutaneous tissues of the American black bear (*Ursus americanus*) and Japanese black bear (*Ursus thiabetanus japonicus*) [1]. It is vectored by black flies in many parts of the USA and Japan. The Center for Diseases Control and Prevention in the USA has suggested that *D. ursi* has the potential to subsequently infect humans [2]. *D. ursi* was shown to parasitize the subscapular connective tissue and perirenal adipose tissue of a black bear in Tamba district, Hyogo Prefecture, in the western part of Japan [3]. The morphology of *D. ursi* has been studied extensively and described in detail [4]. We also identified the same nematode female and male worms parasitizing the cervical subfascia of a black bear in Miyama district, near Tamba district, Kyoto Prefecture, Japan.

The present human case occurred in Shirakawa city, Fukushima Prefecture, in the northern part of Japan, in which *D. ursi* infection has not been detected in black bears even though black bears frequently appear around this area and school children carry bells to scare wandering bears.

The Center for Diseases Control and Prevention in the USA described the life cycle of *Anisakis*, a nematode that parasitizes the gastrointestinal mucosa of humans. The paratenic hosts of anisakiasis are marine fishes, with salmon now being the main infective source for humans in Japan [5]. Salmon is regularly consumed in Japan. We have the recent data that all *anisakis* species collected from salmons: The collected worms from *Oncorhynchus keta*, chum salmon (11 positive/11 examined); *Oncorhynchus masou*, masu salmon (2/2); *Oncorhynchus gorbuscha*, pink salmon (2/2); and *Oncorhynchus nerka*, Sockeye salmon (5/5) were *Anisakis simplex* sensu stricto (s.s.) larvae, comparing with those from mackerel (28 positive/53 examined). Although more than 2000 gastrointestinal cases occur every year [6, 7], extra-gastrointestinal anisakiasis is less common, with only approximately 60 cases being reported to date [6, 8]. A case of *Anisakis pegreffii*, one of the siblings of *A. simplex*, was molecularly described from archival paraffin sections of Italian and Croatian patients [9, 10]. We herein reported a combined case of *D. ursi* infection and granulomatous *A. simplex* s.s. infection based on tissue sections.

## Case presentation

The patient was an 83-year-old Japanese female. She previously worked as a farmer and lived by herself in Shirakawa city, Fukushima Prefecture. Subcutaneous parasitic granulomas were present on the left abdomen (near the navel) and left scapula with itching when admitted to the hospital. A pathological examination of surgically dissected tissue sections from each region revealed eosinophilic granulomas containing different species of parasites. One month later, the patient was admitted to the hospital with fever, weight loss, diarrhea, and vomiting. She had systemic lymphadenopathy and hepatosplenomegaly. She was tentatively diagnosed with diffuse large B cell lymphoma based on bone marrow biopsy findings. She died due to the systemic metastasis of lymphoma (without any parasitic granulomas appearing on her skin) approximately 2 weeks after her admission.

In order to enable the morphological and molecular identification of these parasites, morphological check points were compared in the worms appeared in subcutaneous tissues in detail and reexamined genetically. For molecular identification, the total DNA was extracted from paraffin-embedded sections using DEXPAT reagent (Takara Japan) and QIAmp DNA Mini Kit (Qiagen GmbH, Germany). The ITS region (internal transcribed spacer 1) (ITS1, 5.8S rRNA, and ITS2) and mitochondrial DNA region of COX2 of *Anisakis* were amplified by PCR using the extracted *Anisakis* larvae and sequenced [11]. Each of the primers used for PCR amplification of *Anisakis* DNA was 5′-TGAACCTGCGGAAGGATCA-3 (forward) and 5′-CGGGTAATCACGACTGAGCT-3′ (reverse) for ITS1-5.8S rRNA-ITS2 region (700 bp) and 5′-TCAGGATTTTGGTTTGATGTTT-3′ (forward) and 5′-ATTCTCCATAAAACCTATACAC-3′ (reverse) for COX2 region (682 bp). The mixture was denatured at 94 °C for 3 min, followed by 40 cycles at 94 °C for 30 s, 48 °C for 40 s, and 72 °C for 50 s, with final extension at 72 °C for 7 min on a thermocycler (GeneAmp PCR System 9700; Applied Biosystems, Foster City, CA, USA) for ITS and COX2 products. Electrophoresis confirmed the 700-bp PCR product of ITS region and 682-bp PCR product of COX2 of *Anisakis* DNA amplification.

The 5S rDNA sequence of *Dirofilaria* species was amplified and analyzed by PCR using different four reverse primer sets with the same former primer set for the species identification of *D. ursi*. The selected primers used for PCR amplification of *Dirofilaria* species was forward (5′-TGGGCCTGGTTAGTACTTGG-3′) and reverse (5′-GGGCCGTAACATTCAGTCAG-3′) primers for 5S rRNA. The mixture was denatured at 94 °C for 3 min, followed by 40 cycles at 94 °C for 30 s, 63 °C for 40 s, and 72 °C for 50 s, with final extension at 72 °C for 7 min on a thermocycler for 5S rRNA product (187 bp). Electrophoresis confirmed the 187-bp PCR product of *Dirofilaria* DNA amplification. The amplified polymerase chain reaction (PCR) products were examined and compared with DNA databases. A molecular analysis was conducted on the COX2 of *Anisakis* and 5S rRNA sequences of *Dirofilaria*. PCR products of a 5S rRNA of *D. ursi* were directly sequenced and also cloned in a pGEM-T easy vector (Promega) containing the T7 and SP6 promoter sequences around multiple cloning sites according to the manufacturer’s protocol. The plasmid-cloned 187-bp PCR product was purified using a GenEluteTM HP Plasmid Miniprep kit (Sigma-Aldrich). After purification, the plasmid was sequenced using T7 promoter (5′-TAATACGACTCACTATAGG-3′) and SP6 promoter (5′-ATTTAGGTGACACTATAGAA-3′) primers and a BigDye Terminator v3.1 cycle Sequencing kit (Applied Biosystems) following the manufacturer’s protocols. Sequences were obtained using an ABI Genetic Analyzer (Applied Biosystems). As a result, we obtained full-length sequences of the cloned PCR products. Sequences were assembled and aligned using Genetyx version 11 (Genetyx Corporation) [12]. Sequence regions were identified using BLAST searches and comparisons with the sequences of *D*. *ursi* and *Dirofilaria immitis*, which had been deposited in the GenBank database (GenBank accession nos. GQ241944, GQ241942, GQ241943, GQ241945, FJ874773.1, and EU360964). The morphological definitions of the two parasitic granulomas were analyzed in H&E sections. The ventral subcutaneous granuloma was 46 × 20 × 25 mm in size, while the dorsal subcutaneous granuloma was 28 × 15 × 15 mm.

**Fig. 1:**
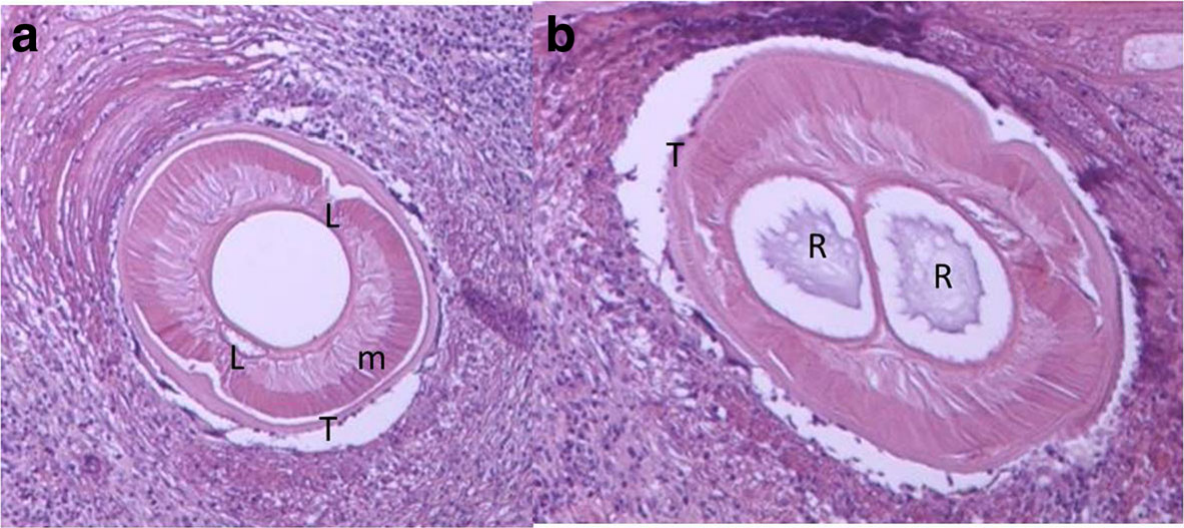
**a**, **b** Paraffin section of *Anisakis* larva in a ventral subcutaneous lesion. m muscle, L lateral chord, R rennet cell, T transverse striation

**Fig. 2:**
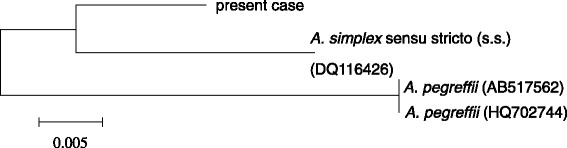
Phylogenetic analysis of *Anisakis* species based on COX2 sequences (532 bp). Nucleotide sequences were aligned and phylogenetic tree analysis was conducted using the Genetyx ver 11 software

Cross sections of the larva of the nematode *Anisakis* were detected in ventral subcutaneous tissue (Fig. 1a, b). These sections showed *Anisakis* larva based on the morphological appearance of the structures of the cuticle, muscle, lateral chord, rennet cell, and transverse striations. PCR was conducted for the COX2 and ITS1-5.8S rRNA-ITS2 regions of *Anisakis* larva. The ITS sequences showed close similarity in the siblings such as *A. simplex* s.s. and *A. pegreffii*. So, COX2 gene was chosen as the target sequence in the reason why COX2 region of mtDNA is well known to be less than ITS1-5.8S rRNA-ITS2 region of nuclear DNA in the base substitution. The regions of COX2 were identified using BLAST searches and comparisons with the sequences of *A. simplex* s.s. and *A. pegreffii*, which had been deposited in the GenBank database (GenBank accession nos. AB517562, HQ702744, and DQ116426). Multiple sequence alignment was carried out using the Genetyx ver 11 software. A genetic analysis of the DNA sequence from the nucleotide COX2 sequences (532 bp, partial sequence) of this *Anisakis* species was similar to that of *A. simplex* s.s. but different from that of *A. pegreffii* with 98.5% identity to *A. simplex* s.s. and 92.2% identity to *A. pegreffii* at 8 positions. The phylogenetic tree based on COX2 sequences analysed by Genetyx ver 11 software revealed that this specimen belongs to *A. simplex* s.s. (Fig. 2).

**Fig. 3:**
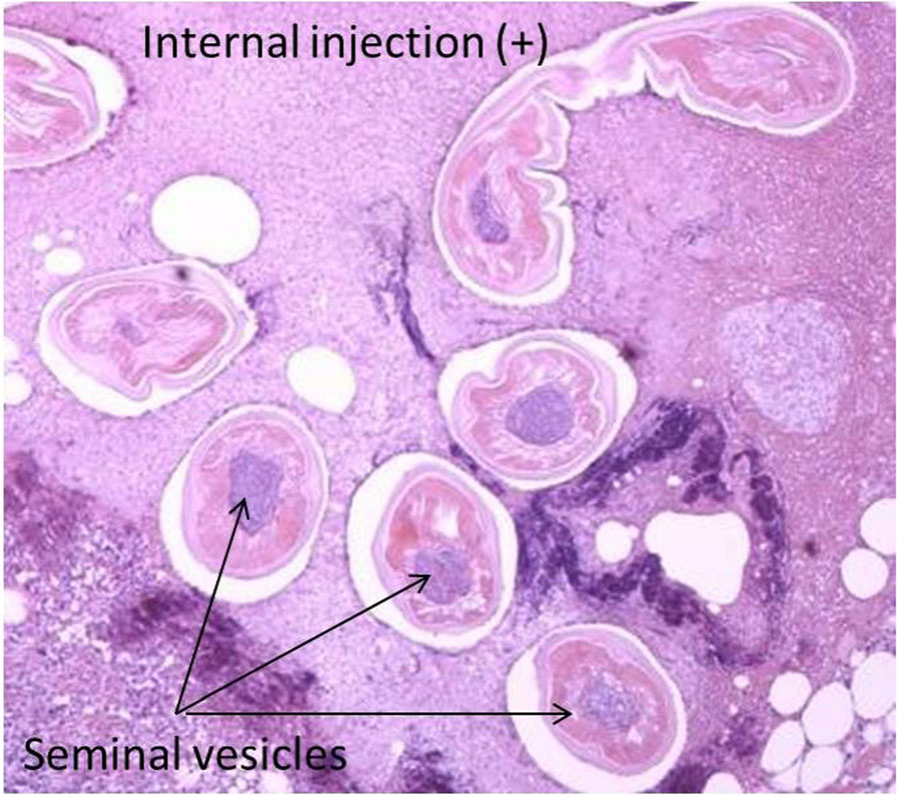
Paraffin section of *Dirofilaria* species in a dorsal subcutaneous lesion

**Fig. 4:**
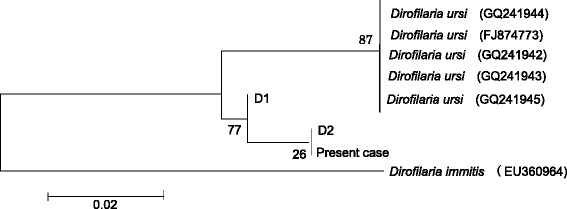
Phylogenetic analysis of *Dirofilaria* species based on 5S rRNA sequences (90 bp). Nucleotide sequences were aligned using the Genetyx ver 11 and CLC Sequence Viewer 6 software. Phylogenetic analyses were conducted using the Mega 6 software. Genetic relationships were inferred by the neighbor-joining (NJ) method. D1 and D2 means *Dirofilaria ursi* worms collected from Japanese bears in Gifu Prefecture in the middle part of Japan

Cross sections of the nematode *Dirofilaria* species were detected in dorsal subcutaneous tissue (Fig. 3). The present case was diagnosed as dirofilariasis due to male adults because of the presence of seminal vesicles in the center of the section. *Dirofilaria* species were 200–360 μm in diameter (body width) (Table 1). There were 65–82 longitudinal ridges (Table 1). The distance between longitudinal ridges was 5–12.5 μm (Table 1). PCR amplification with primers directed to the mitochondrial 16S rRNA region of *D. immitis* [13] and COX1 region of *Dirofilaria repens* [14] and COX1 region of *Onchocerca dewittei japonica* [15] was conducted. PCR products of the *D. immitis* 16S rRNA, *D. repens* COX1, and *O. dewittei japonica* COX1 regions were all negative. The diameter, cuticle thickness, number of longitudinal ridges, and distance between longitudinal ridges on the surface of the present *Dirofilaria* species in human dorsal lesions are summarized in Table 1. These results indicated that the present *Dirofilaria* species was very similar to *D. ursi*. PCR amplification with primers directed to the mitochondrial 5S rRNA of *D. ursi* was conducted. The PCR product of *Dirofilaria* species was amplified using four different reverse primers, with the same primers being used for the species identification of *D. ursi*. A genetic analysis of DNA sequences from the nucleotide 5S rRNA region (95 bp, partial sequence) of this *Dirofilaria* species revealed 97.4% identity to *D. ursi* and 89.3% to *D. immitis*. Moreover, sequences obtained by the TA cloning of PCR products (187 bp, partial) from this *Dirofilaria* species showed 98% identity to *D. ursi* and 84% identity to *D. immitis*. A phylogenetic relationship according to the 5S rRNA gene (90 bp, partial sequence) was conducted, and the present *Dirofilaria* worm was very similar to *D. ursi* (GenBank accession nos. GQ241944, GQ241942, GQ241943, GQ241945, FJ874773.1, and EU360964). The phylogenetic tree of this *D. ursi* containing worms collected from Japanese black bears in Gifu (the middle part of Japan) based on 5S rRNA sequences is shown in Fig. 4.

**Table 1:** Summary of the width and number of longitudinal ridges on the surface of *Dirofilaria* species in a dorsal lesion

Section	Diameter (μm)	Thickness of cuticle (μm)	Longitudinal cuticular ridges	
			Number	Distance (μm)
1	200–270	15	77	5
2	250–310	12.5	70	10
3	240–280	15	69	10
4	250–320	10	72	10
5	250–310	12.5	72	12.5
6	230–360	15	71	7.5
7	250–315	17.5	82	10
8	200–320	12.5	65	10
9	270–320	7.5	71	7.5
10	220–310	12.5	72	7.5

## Discussion and conclusions

We herein present a combined human case of *D. ursi* infection in dorsal subcutaneous tissue and a granulomatous *A. simplex* s.s. infection in ventral subcutaneous tissue. These subcutaneous parasitic lesions were operated and removed but had not been recognized these subcutaneous nodules until operation because of living by herself and nobody detected. Anisakiasis is one of the most important zoonoses reported worldwide every year, particularly in countries in which eating raw fish is a custom. More than 2000 cases of anisakiasis are reported every year in Japan, and this number has recently increased, possibly due to the custom of eating various raw or undercooked fishes as sashimi or sushi. Additionally, extra-gastrointestinal anisakiasis is less common and caused by a larval *Anisakis* nematode which escaped the gastrointestinal wall and makes a lesion at the abdominal wall and cavity, abdominal omentum, mesentery, and subcutaneous tissues, with only approximately 60 cases being reported to date in Japan. The pathogenic potential of two sibling nematodes, *A. simplex* s.s. and *A. pegreffii* derived from living larvae or larvae in tissue sections, was demonstrated based on in vitro penetration ability, acid tolerance, and in vivo experimental infection studies [16, 17]. This case was finally found to be *Anisakis* species similar to *A. simplex* s.s. using a DNA analysis of tissue sections*.* It is important to clearly differentiate between *D. ursi* and *D. immitis* because of their similar morphologies. The body width of the midbody of *D. ursi* was 380–520 μm, and the distance between longitudinal ridges was 9–14 μm. Furthermore, *Dirofilaria* has to be morphologically differentiated from filarial *Onchocerca* species in cross sections of subcutaneous tissues because both are causative agents in subcutaneous nodules and vectored by black flies, while their final hosts are different. The body width of *O. dewittei japonica* collected from wild boars was previously reported to be 85–135 μm [18–21]. The cuticle of *Dirofilaria* species (final host: wild boar) has internal projections, whereas *O. dewittei japonica* does not. The thickness of the cuticle of *D. ursi* was very thin, whereas that of *O. dewittei japonica* was thick and composed of 4–5 layers. The number of longitudinal ridges was lower in *D. ursi* than in *O. dewittei japonica*. The longitudinal ridges of *O. dewittei japonica* were shorter and more prominent than those of *D. ursi*. The number of longitudinal ridges in *O. dewittei japonica* was 138–152, whereas that in *D. ursi* was 62. In conclusion, a molecular analysis of the COX2 and 5S rRNA sequences of each worm revealed that the *Anisakis* larva found in the ventral region belonged to *A. simplex* s.s. and the male *Dirofilaria* found in the dorsal region was diagnosed as *D. ursi*.

The present case showed a combined human case of *D. ursi* and *A. simplex* s.s. infections. The results of this study will contribute to the identification of unknown parasites appeared in histological sections. Additionally, a recent molecular analysis showed that this *D. ursi* was very similar to one of the *D. ursi* collected from a Japanese black bear (captured in Gifu Prefecture in the middle part of Japan). The difference between Japanese *D. ursi* and American *D. ursi* was 3–4%.
